# Use of bone-modifying agents for bone metastases from neuroendocrine neoplasms: a single-centre audit

**DOI:** 10.1530/EC-26-0252

**Published:** 2026-10-26

**Authors:** Wessam Osman, Preetha Aravind, Hemanth Prabhudev, Jeannie Todd, Ramona Nistor, Antonio D’Alessio, Rohini Sharma, Tami Grunewald, Florian Wernig

**Affiliations:** ^1^ Department of Endocrinology, Imperial College Healthcare NHS Trust, London, UK; ^2^ National Diabetes & Endocrine Centre, The Royal Hospital, Muscat, Oman; ^3^ Department of Oncology, Imperial College Healthcare NHS Trust, London, UK; ^4^ Division of Diabetes, Endocrinology and Metabolism, Imperial College London, London, UK; ^5^ Department of Surgery and Cancer, Imperial College London, London, UK

**Keywords:** neuroendocrine neoplasms, bone metastases, bone-modifying agents, denosumab, zoledronic acid, skeletal-related events

## Abstract

Bone metastases in neuroendocrine neoplasms are uncommon but cause significant morbidity. A recent Italian survey of 320 patients with neuroendocrine neoplasms and bone metastases reported skeletal-related events in 23% and bone-targeted agent use in 31%, highlighting limited evidence for bone-modifying agents. We identified adults with confirmed or clinically suspected neuroendocrine bone involvement who received a bone-modifying agent at a UK tertiary centre (January 2019–September 2024), using institutional prescribing records. Demographic, tumour, treatment, and toxicity data were extracted from records. Twenty-two patients received treatment (denosumab 17 (77%); zoledronic acid 5 (23%)). Most (91%) had grade 1–2 tumours; 64% had gastroenteropancreatic primaries. Bone metastases were synchronous in 45% and widespread (more than three lesions) in 73%. At diagnosis, 23% were symptomatic; during follow-up, 77% developed pain, high blood calcium, or a skeletal-related event. Skeletal-related events, a composite of pathological fracture, spinal cord compression, radiotherapy to bone, or orthopaedic surgery, occurred in 41% (radiotherapy alone in 36%); pain-relieving medication was used universally. Treatment was mainly given every 12 weeks (median six doses). Low blood calcium occurred in 9% and osteonecrosis of the jaw in 5%; dental assessment before therapy was recorded in only 9%. Treatment use was heterogeneous, reflecting a lack of disease-specific guidelines, and recorded toxicity was infrequent and generally manageable, although exposure varied s. Our findings highlight the need for prospective studies and structured registries to clarify treatment use, dosing, and impact on quality of life and prognosis.

## Introduction

Bone metastases (BMs) from neuroendocrine neoplasms (NENs) are relatively uncommon but clinically important. Large series estimate a prevalence of 4–12% overall, with rates varying by primary tumour site and grade (1, 2). Multi-institutional analyses suggest bone involvement in 12% of NENs, including 8% of pancreatic neuroendocrine tumours (NETs), 9% of midgut tumours, 20% of high-grade neuroendocrine carcinomas (NECs), and up to 25% of phaeochromocytomas/paragangliomas (3). In a recent Italian nationwide series of 320 patients with NEN-related BMs, BMs occurred in fewer than 15% of all NENs but represented the first site of metastatic spread in 41% of patients (4). BMs are typically a late manifestation linked to more aggressive pathology. Patients with synchronous BMs – occurring within six months of initial diagnosis – carry poorer prognostic features (higher Ki-67 and greater tumour burden) compared with patients with metachronous BMs (5, 6).

Clinically, BMs may cause pain and hypercalcaemia and may result in skeletal-related events (SREs), conventionally defined as pathological fracture, spinal cord compression, radiotherapy to bone, or orthopaedic surgery/stabilisation involving bone with substantial impact on quality of life and healthcare utilisation (5). This distinction is important because pain is a symptom of skeletal involvement rather than an SRE, while hypercalcaemia may be bone-related or humoral, including through PTH-related peptide secretion. Although NEN-related BMs appear to be mostly osteoblastic/sclerotic, a mixed blastic–lytic phenotype is common in higher-grade tumours, reflecting pathobiological heterogeneity and further complicating management (7, 8, 9).

In contrast to breast or prostate cancer, where randomised trials established bone-modifying agents (BMAs; intravenous bisphosphonates or the RANKL inhibitor denosumab) as standard treatment to reduce skeletal morbidity, there is no NEN-specific consensus on BMA use (7). Current international guidance provides limited direction, and practice varies widely, often extrapolating clinical practice from other solid tumours (3, 7).

We conducted a single-centre retrospective audit to describe real-world patterns of BMA use for NEN-related BMs, associated toxicities, and supportive interventions and to highlight opportunities for practice improvement and prospective evaluation.

## Materials and methods

This was a single-centre, retrospective audit at a UK tertiary referral centre. This project was registered with local clinical governance; given its retrospective service evaluation nature, research ethics committee approval and individual patient consent were not required.

The audit identified patients from institutional denosumab and zoledronic acid prescribing/administration records between January 2019 and September 2024. Records were cross-referenced with the electronic health record (Cerner, Oracle Health, USA). Eligible neoplasms comprised histologically confirmed well-differentiated NETs (WHO G1–G3) and paragangliomas with clinically documented, imaging-supported bone involvement. Poorly differentiated neuroendocrine carcinomas were not represented in the cohort. Patients receiving a BMA solely for osteoporosis or another non-metastatic indication were excluded unless contemporaneous clinical documentation also supported metastatic skeletal involvement. Bone involvement was accepted when documented on specialist-reviewed cross-sectional or functional imaging and/or in the treating multidisciplinary team record. Patients with unknown primary were included if NEN histology was confirmed elsewhere. Data on demographics, tumour characteristics, primary site, grade, functional status, timeline of disease (synchronous versus metachronous), and disease distribution (widespread, defined as >3 bony lesions) were collected. Further data collection included type of BMA, dose, and schedule; initiating speciality (endocrinology versus oncology); concomitant treatments, including analgesia and external beam radiotherapy (EBRT); dental pre-assessment; and outcomes/toxicities. SREs were defined as pathological fracture, spinal cord compression, radiotherapy to bone, or orthopaedic surgery/stabilisation involving bone. Pain and hypercalcaemia were captured separately and were not classified as SREs. Key outcomes were toxicities attributable to BMAs, specifically hypocalcaemia and osteonecrosis of the jaw (ONJ). Follow-up extended from first BMA administration to death or last clinical contact, and available records were censored at the time of audit extraction. Patients were identified from BMA prescribing/administration records rather than from an all-NEN registry; consequently, this audit was designed to describe treated patients and was not intended to estimate the prevalence of bone metastases among all patients with NENs or to compare treated and untreated patients.

Given the small sample size (*n* = 22), analyses were descriptive (counts and percentages). Continuous variables are summarised as mean (SD) or median (IQR), as appropriate; categorical variables are presented as *n* (%). Treatment exposure is reported as treatment duration and the number of BMA administrations where available. No comparative or inferential analyses were undertaken because the audit did not include an untreated comparator cohort.

## Results

We identified 22 patients who received a BMA in the context of confirmed or clinically suspected NEN-related bone involvement. The mean age was 63 years (range: 29–84). Fourteen (64%) were male, and eight (36%) were female. Fourteen (64%) had gastroenteropancreatic primaries; eight (36%) had other sites (paraganglioma (4), lung (3), and unknown primary (1)). All tumours were well-differentiated NETs or paragangliomas; poorly differentiated NECs were not represented. Most tumours were G1–G2 (20/22, 91%); two (9%) were G3. BMs were synchronous at diagnosis in 10/22 (45%) and metachronous in 12/22 (55%). Widespread bone disease (>3 lesions) was present in 16/22 (73%).

At initial BM diagnosis, 5/22 (23%) were symptomatic; ultimately, 17/22 (77%) developed skeletal symptoms during follow-up. Where symptom subtype was documented (*n* = 11), pain was most frequent (7/11, 64%), followed by hypercalcaemia (4/11, 36%). Using the conventional composite definition of pathological fracture, spinal cord compression, radiotherapy to bone, or orthopaedic surgery/stabilisation involving bone, SREs were identified in 9/22 patients (41%). EBRT was delivered to 8/22 (36%), and one additional patient had malignant spinal cord compression. Pain and hypercalcaemia were analysed separately and were not included in the SRE composite. Analgesics were used universally.

Denosumab was used in 17/22 (77%) – typically 120 mg with approximately 12-weekly dosing intervals – while 5/22 (23%) received zoledronic acid (ZA) 4 mg, most commonly on a 12-weekly schedule (4/5, 80%). One patient received denosumab 60 mg at similar intervals (grade 1 NETs), and one received monthly ZA. Initiation of BMAs was evenly split between oncology (11/22, 50%) and endocrinology (11/22, 50%). The recorded indication for BMA initiation was the presence of bone involvement in the treated cohort; more granular indication categories, including pain, progression, hypercalcaemia, or prophylaxis in extensive bone disease, could not be reconstructed reliably from the aggregate audit sheet and are therefore not reported.

The median interval from recorded BM diagnosis to first BMA administration was 97 days (IQR: 30–396; range: 0–4,868 days), approximately 3.2 months (IQR: 1.0–13.0 months). The extreme upper value reflected an approximate year-only date in one historical record. The median number of BMA administrations was 6 where recorded, while treatment duration ranged from a single administration to 36 months. Using death date or audit extraction censoring where a later last contact date was not available, the median follow-up from first BMA administration was approximately 11.4 months (IQR: 6.0–28.1). Therapy was changed due to progression in 2/22 (9%). Ten patients (45%) died during follow-up, all due to progressive NENs.

Documented toxicities included hypocalcaemia in 2/22 (9%) and ONJ in 1/22 (5%); no renal toxicity was recorded. The patient with ONJ had received denosumab 120 mg SC, with nine completed 120 mg administrations recorded between September 2020 and May 2022. Denosumab was subsequently held because of dental issues and later resumed in March 2024; ONJ was documented during follow-up. Calcium/vitamin D supplementation and laboratory monitoring were incompletely documented. Of the hypocalcaemia cases identified on review, one had concurrent vitamin D insufficiency (38.4 nmol/L), whereas another had previously replete vitamin D values but incomplete contemporaneous testing. Dental pre-assessment was clearly documented in 2/22 (9%); documentation was absent or uncertain in the remaining patients.

Table 1 summarises baseline characteristics and outcomes.

**Table 1:** Tumour characteristics and outcomes in patients with neuroendocrine neoplasm-related bone metastases (*n* = 22).

Characteristics	Patients, *n* (%)
Type of BMA	
Denosumab	17 (77)
Zoledronic acid	5 (23)
Primary tumour site	
Gastroenteropancreatic	14 (64)
Other sites	8 (36)
Tumour grade	
Grade 1–2	20 (91)
Grade 3	2 (9)
Timing of BM at diagnosis	
Synchronous	10 (45)
Metachronous	12 (55)
Distribution of bone metastases	
Widespread (>3 lesions)	16 (73)
≤3 lesions	6 (27)
Symptoms at BM diagnosis	5 (23)
Symptoms developing later	
Pain (*n* = 11)	7 (64)
Hypercalcaemia (*n* = 11)	4 (36)
SREs (composite: *n* = 22)	9 (41)
Additional management	
Radiotherapy to bone (EBRT)	8 (36)
Dosing frequency (12 weekly)	
Denosumab	15 (88)
Zoledronic acid	4 (80)
Treatment exposure	
Median 6 administrations	1–36 months
BMA change due to progression	2 (9)
Toxicities	
Hypocalcaemia	2 (9)
ONJ	1 (5)
No recorded renal toxicity	0
Dental assessment documented	2 (9)
Deaths (due to progressive NEN)	10 (45)

BMA, bone-modifying agent; BM, bone metastasis; EBRT, external beam radiotherapy; NEC, neuroendocrine carcinoma; NEN, neuroendocrine neoplasm; NET, neuroendocrine tumour; ONJ, osteonecrosis of the jaw; PRRT, peptide receptor radionuclide therapy; SRE, skeletal-related event; ZA, zoledronic acid.

## Discussion

This single-centre audit provides insights into the contemporary use of BMAs for NEN-related BMs and documents substantial variability in agent selection, dosing interval, and timing of initiation, mirroring the heterogeneous diagnostic and therapeutic strategies reported in the Italian nationwide survey (4). Three observations emerge: first, most patients ultimately experienced skeletal symptoms despite only a minority being symptomatic at the time bone disease was first identified, underscoring the morbidity of bone involvement in NENs and the need for a defined supportive treatment pathway. Second, extended-interval schedules (approximately 12-weekly) were frequently adopted to lessen treatment burden; while such intervals are supported for ZA in other tumour types, evidence specific to NENs, and to interval extension with denosumab, remains limited. Third, recorded adverse events included hypocalcaemia and ONJ, but toxicity frequencies should be interpreted cautiously given the small cohort size, variable exposure, and retrospective ascertainment. The low rate of clearly documented dental pre-treatment assessment represents a remediable quality care–process gap.

Among NEN patients with bone metastases, published estimates of SRE frequency vary substantially depending on tumour type, cohort selection, definition of SRE, observation time, and exposure to bone-targeted therapies (4, 7, 10, 11, 12). The Italian nationwide cohort reported SREs excluding pain in 23% of 320 patients and found SREs to be independently associated with shorter overall survival (4). These estimates are not directly comparable with our BMA-treated audit cohort because of differences in case selection, event definitions, observation time, and treatment exposure. Using a conventional composite definition that includes radiotherapy to bone, ascertainable SREs were recorded in 9/22 patients (41%) in this series. No inference regarding a protective effect of BMAs on SRE incidence or survival can be made.

The choice between ZA and denosumab in NENs is typically pragmatic and informed by comorbidity, logistics, and clinician preference. ZA benefits from long-term experience and cost-effectiveness but requires renal monitoring. Denosumab avoids renal dose adjustment and is convenient to administer; however, planned discontinuation strategies are essential given rebound bone turnover and hypocalcaemia risk. Our dataset is not powered to compare efficacy or safety between agents, and no agent-specific recommendations can be made. In the Italian nationwide survey, only 31% of patients received a bone-targeted agent, predominantly bisphosphonates, and timing of initiation varied widely (4). This heterogeneity, together with the pragmatic use of both agents in our cohort, supports the call for objective, risk-adapted criteria to guide when and how to start BMAs in NENs. The audit was not designed to estimate BMA effectiveness; the absence of an untreated comparator and likely confounding by indication preclude inference that BMA exposure altered SRE risk.

The optimal time to commence BMAs in NENs remains unresolved. A risk-adapted approach appears reasonable: immediate initiation may be considered for symptomatic lesions, diffuse or multifocal bone disease, or radiological disease progression, while solitary or indolent lesions may be managed with close surveillance and a low threshold to start treatment at the earliest sign of progression or pain. Importantly, BMAs should be positioned as an adjunct to local measures rather than as a substitute. EBRT provides pain palliation and local control for focal disease, and timely orthopaedic assessment may avert catastrophic structural events in selected patients. Fazio *et al.* identified primary lung site, Ki-67 ≥ 55%, >10 BMs, mixed osteoblastic/osteolytic pattern, prior lung metastases, and SREs as adverse prognostic factors for survival after BM diagnosis, and Ki-67 ≥ 55% and non-bone-first metastases as predictors of SREs (4). Incorporating such variables into risk assessment may help prioritise early BMA consideration and intensive surveillance for those at highest risk of skeletal morbidity.

The low rate of clearly documented dental review prior to BMA exposure highlights an opportunity for service improvement. A standardised care pathway could include i) dental assessment and optimisation before first dose; ii) baseline and periodic monitoring of calcium, vitamin D, and renal function, with routine supplementation where indicated; iii) explicit triggers for radiotherapy and orthopaedic referral; and iv) scheduled treatment reviews to individualise duration, dose interval, and discontinuation planning.

The strengths of this audit include real-world capture across endocrinology and oncology services and detailed documentation of dosing patterns and supportive care processes. Limitations include the small sample size, single-centre design, BMA-based sampling frame, absence of a non-BMA comparator cohort, and retrospective ascertainment, all of which limit inference on clinical effectiveness. The study population represents patients exposed to a BMA and should not be interpreted as the complete institutional population with NEN-related bone metastases. One retained case had possible spinal metastases with non-diagnostic imaging, reflecting the uncertainty sometimes encountered in retrospective real-world audit datasets. Symptom sub-classification, pain response, vitamin D supplementation, and dental assessment documentation were incomplete. Exact event dates were not consistently available for all SRE components, limiting differentiation between events occurring before and after BMA initiation. Pain response could not be assessed consistently and was confounded by concurrent analgesia, radiotherapy, and anticancer treatment. Toxicity frequencies should be interpreted cautiously because follow-up duration, treatment duration, and cumulative BMA exposure varied substantially; this is particularly relevant to ONJ, a cumulative toxicity that may be underestimated in cohorts with short or variable exposure. The predominance of G1–G2 well-differentiated tumours and absence of poorly differentiated NECs limit generalisability to high-grade disease, in which the natural history, competing mortality, and skeletal-related event risk may differ substantially. These constraints reinforce the need for prospective evaluation.

Clinical implications are two-fold. First, BMA use in NEN-related BMs is variable in real-world practice and should be embedded within a multidisciplinary pathway that includes bone health optimisation, dental surveillance, and explicit toxicity monitoring. Second, until NEN-specific evidence is available, harmonising local practice around a pragmatic risk-adapted algorithm and agreed prospective follow-up metrics may reduce unwarranted variation and improve patient-centred outcomes.

## Conclusion

In this tertiary-centre series, BMA selection, initiation, and dosing schedules for NEN-related bone involvement varied substantially, with extended-interval schedules commonly employed. Recorded adverse events included hypocalcaemia and ONJ, although their frequency is difficult to interpret because follow-up and cumulative exposure differed between patients. Pain and hypercalcaemia should be separated from conventionally defined SREs, and no inference regarding BMA effectiveness can be made from this treated cohort without an untreated comparator. Standardised dental assessment, calcium and vitamin D optimisation, explicit treatment indications, scheduled treatment review, and prospective recording of skeletal outcomes should be incorporated into local care pathways while definitive NEN-specific data are awaited. Collaborative initiatives, such as the Italian nationwide survey and the MONET observational programme, should prioritise the timing of BMA initiation, agent and schedule selection, discontinuation strategies, and patient-reported outcomes to inform future consensus guidance (4).

## Declaration of interest

AD reports educational support for congress attendance from Roche, AstraZeneca, and Novartis; consultancy fees from Roche, Chugai, and AstraZeneca; and speaker fees from Roche, AstraZeneca, Eisai, and Chugai. All other authors declare no competing interests.

## Funding

This work did not receive any specific grant from any funding agency in the public, commercial, or not-for-profit sector.

## Author contribution statement

FW and TG conceived the study; FW, TG, and RS designed the methodology; WO, PA, HP, and RN curated the data; all authors performed formal analysis; WO and PA wrote the original draft; all authors reviewed and edited the manuscript; FW supervised the study.

## Data availability

The de-identified dataset underlying this audit is available from the corresponding author on reasonable request; institutional policies preclude public deposition of patient-level data.

## Ethics/consent statement

This retrospective service evaluation was registered with the local clinical governance office at Imperial College Healthcare NHS Trust. Given the evaluation design and use of de-identified data, research ethics committee approval and individual patient consent were not required.

## References

[1] Altieri B, Di Dato C, Martini C, et al. Bone metastases in neuroendocrine neoplasms: from pathogenesis to clinical management. Cancers 2019 11 1332. (10.3390/cancers11091332) PMC6770134 31500357

[2] Guan M, He I, Luu M, et al. Palliative radiation therapy for bone metastases in neuroendocrine neoplasms. Adv Radiat Oncol 2019 4 513–519. (10.1016/j.adro.2019.03.014) PMC6639761 31360808

[3] Van Loon K, Zhang L, Keiser J, et al. Bone metastases and skeletal-related events from neuroendocrine tumors. Endocr Connections 2014 4 9–17. (10.1530/ec-14-0119) PMC4285767 25430999

[4] Fazio N, Maisonneuve P, Frezza AM, et al. Bone metastases from neuroendocrine neoplasms: results of an Italian nationwide survey of natural history and management. J Neuroendocrinol 2025 38 e70115. (10.1111/jne.70115) PMC12799322 41255062

[5] Scopel M, De Carlo E, Bergamo F, et al. Bone metastases from neuroendocrine tumors: clinical and biological considerations. Endocr Connections 2022 11 e210568. (10.1530/ec-21-0568) PMC9254287 35671272

[6] Alexandraki KI, Pizanias M, Uri I, et al. The prognosis and management of neuroendocrine neoplasms-related metastatic bone disease: lessons from clinical practice. Endocrine 2019 64 690–701. (10.1007/s12020-019-01838-8) 30635793

[7] Garcia-Torralba E, Spada F, Lim KHJ, et al. Knowns and unknowns of bone metastases in patients with neuroendocrine neoplasms: a systematic review and meta-analysis. Cancer Treat Rev 2021 94 102168. (10.1016/j.ctrv.2021.102168) 33730627

[8] Lavezzi E, Jaafar S, Chiardi I, et al. Clinical presentation of bone metastases in neuroendocrine tumors: a retrospective cohort study. Endocr Relat Cancer 2025 32 e250147. (10.1530/erc-25-0147) 40919687

[9] Cives M, Rizzo F, Simone V, et al. Reviewing the osteotropism in neuroendocrine tumors: the role of epithelial-mesenchymal transition. Neuroendocrinology 2016 103 321–334. (10.1159/000438902) 26227818

[10] Wedin M, Janson ET, Wallin G, et al. Impact on symptoms and survival of bone metastases in patients with small-intestinal neuroendocrine tumours. J Neuroendocrinol 2025 37 e70073. (10.1111/jne.70073) PMC12488365 40778558

[11] Mathew A, Fendler WP, Theysohn J, et al. Bone metastases in patients with pancreatic NETs: prevalence and prognosis. Horm Metab Res 2023 55 827–834. (10.1055/a-2159-5548) 37611636

[12] Mathew A, Hauptmeier P, Schaarschmidt BM, et al. High incidence and poor prognosis of bone metastases in functioning small intestinal neuroendocrine tumors. Front Endocrinol 2025 16 1680209. (10.3389/fendo.2025.1680209) PMC12588865 41210494

